# Supplementary material for: Systemic delivery of full-length dystrophin in Duchenne muscular dystrophy mice
Source: Nat Commun. 2024 Jul 21;15:6141. doi: 10.1038/s41467-024-50569-6 (PMC11271493; doi:10.1038/s41467-024-50569-6)
Supplement: Supplementary file 1 — Supplementary Information [file 41467_2024_50569_MOESM1_ESM.pdf]

## Supplementary Information

# Systemic Delivery of Full-Length Dystrophin in Duchenne Muscular Dystrophy Mice

**First author's surname**: Zhou

**Short title**: Full-Length Dystrophin Gene Therapy

Yuan Zhou[1], Chen Zhang[1], Weidong Xiao[1], Roland W. Herzog[1], Renzhi Han[1,*]

**Supplementary information includes:**

**Supplementary Table 1**. List of plasmids used in this study.

**Supplementary Table 2**. List of primers used in this study.

**Supplementary Figure 1-8**.

**Supplementary Table 1**. List of plasmids used in this study.

| ID | Description |
|---|---|
| **pZC288 (Dys-N1)** | meCMV driven DMD N-terminal fragment fused with Cfa split intein; used in Fig. 1b, Fig. 2, Suppl. Fig. S1, S2 and S3b. |
| **pZC287 (Dys-M1)** | meCMV driven DMD Middle fragment fused with Cfa split intein at the upstream and Gp41-1 split intein at the downstream; used in Fig. 1b, Fig. 2, Suppl. Fig. S1 and S2. |
| **pZC289 (Dys-C1)** | meCMV driven DMD C-terminal fragment fused with Gp41-1 split intein at the upstream; it also carries an intron near the end; used in Fig. 1b, Fig. 2, Suppl. Fig. S1 and S2. |
| **pZC349 (Dys-M2)** | Modified pZC287 with the split site changed from EMP-SSL to IGA-SPT on dystrophin; IGA is mutated to IGY; used in Fig. 2, Suppl. Fig. S1. |
| **pZC350 (Dys-C2)** | Modified pZC289 with the split site changed from EMP-SSL to IGA-SPT on dystrophin; used in Fig. 2b-f, Suppl. Fig. S1a-c. |
| **pZC379 (Dys-C3)** | Modified pZC350 with the intron removed; used in Fig. 2b-l, Suppl. Fig. S1a-f. |
| **pZC380 (Dys-C4)** | Modified pZC379 with the mutation of SPT to SSS and the change of the Kozac sequence from “GCCACCATGAT” to “CAGTTGATGCT”; used in Fig. 2h-l, Suppl. Fig. S1d-f. |
| **pZC382 (Dys-M3)** | Modified pZC349 with the change of Gp41-1$^{N}$ to IMPDH-1$^{N}$ and the mutation of IGA to IGG in dystrophin at the fusion junction; used in Fig. 2n-r, Suppl. Fig. S1g-l, S2 and S3b. |
| **pZC400 (Dys-C5)** | Modified pZC380 with the change of the Kozac sequence from “CAGTTGATGCT” to “GCCACCATGCA” and the addition of a PB29 signal sequence (MHSWNFKLYVM) at the upstream of Gp41-1$^{C}$; used in Fig. 2h-r, Suppl. Fig. S1d-i. |
| **pZC401 (Dys-C6)** | Modified pZC400 with the change of Gp41-1$^{C}$ to IMPDH-1$^{C}$; used in Fig. 2n-r, Suppl. Fig. S1g-l, S2 and S3b. |
| **pZC446 (Dys-C7)** | Modified pZC401 with a polyA regulator A6g inserted before the start codon of the transgene; used in Suppl. Fig. S2. |
| **pZC447 (Dys-C8)** | Modified pZC401 with a polyA regulator A6e2 inserted before the start codon of the transgene; used in Suppl. Fig. S2. |
| **pZC414 (Dys-N1 with Spc5-12)** | Modified pZC288 with the meCMV promoter replaced with Spc5-12; used in Fig. 3, 4, 5, 6, 7 and Suppl. Fig. S4, S5, S6, S7 and S8. |
| **pZC493 (Dys-N-Cat$^{N}$)** | meCMV driven DMD N-terminal fragment fused with atypical split intein Cat; used in Fig. S3b. |
| **pZC495 (Cat$^{C}$-Dys-M-IMPDH)** | meCMV driven DMD Middle fragment fused with Cat split intein at the upstream and IMPDH split intein at the downstream; used in Fig. S3b. |
| **pZC496 (Cat$^{C}$-Dys-M-Vid$^{N}$)** | meCMV driven DMD Middle fragment fused with Cat split intein at the upstream and Vid split intein at the downstream; used in Fig. S3b. |
| **pZC497 (Vid$^{C}$-Dys-C)** | meCMV driven DMD C-terminal fragment fused with VId split intein at the upstream; used in Fig. S3b. |
| **pZC415 (Dys-M3 with Spc5-12)** | Modified pZC382 with the meCMV promoter replaced with Spc5-12; used in Fig. 3, 4, 5, 6, 7 and Suppl. Fig. S4, S5, S6, S7 and S8. |
| **pZC416 (Dys-C6 with Spc2-26)** | Modified pZC401 with the meCMV promoter replaced with Spc2-26; used in Fig. 3, 4 and 5 and Suppl. Fig. S4 and S5. |
| **pZC473 (Dys-C6 with Spc5-12)** | Modified pZC401 with the meCMV promoter replaced with Spc5-12; used in Fig. 5, 6, 47 and Suppl. Fig. S6, S7 and S8. |
| **p37-2iDMD-LR** | Full-length dystrophin cDNA with firefly luciferase and mCherry; used in Fig. 2, Suppl. Fig. S1, S2 and S3. |
| **pZC470 (μ-Dys-v1)** | Spc5-12 driven micro-dystrophin with the deletions of R3-R21 and CT domain; used in Fig. 6,7, Suppl. Fig. S6 and S8. |
| **pZC471 (μ-Dys-v2)** | Spc5-12 driven micro-dystrophin with the deletions of R2-R15, R18-22 and CT domain; used in Fig. 6,7, Suppl. Fig. S6 and S8. |

**Supplementary Table 2**. List of primers used in this study.

| ID | Sequence |
|---|---|
| 453 | TTACTGGTGGAAGAGTTGCC |
| 483 | TCTGAATTCTTTCAATTCGATCCGTAATG |
| 1149 | TTGTTCTGCAAAACCCGCAG |
| 3195 | GGCCTCTAGAGCATGCCCACTCACGGGGATTTCCAAG |
| 3588 | TTCTACTAGTTATTCCTTGACATACAAACAC |
| 3589 | CTGGTTTAGTGAACCGTCAGATCCGCTAGCCACCATGATGCTCAAGAAGATCCTC |
| 3688 | GCAGAGAAAGCCAGTCGGTA |
| 4765 | GATCGCTAGCCACCATGCTTTGGTGGGAAGAAGTAG |
| 4777 | CATTGACTCTCTCCAAGATCA |
| 5187 | GATCGCTAGCCACCATGCTTTGGTGGGAAGAAGTAG |
| 5273 | GGCTAAGCTTCGCTAGCGGATCTGACGGTTCACTAAAC |
| 5586 | GAAATCTAGAAAGTCTCGAGGATGAGAGCAGCCACTACGGGTC |
| 6271 | CAGCTAGCGGATCTGACGGTTCAC |
| 6395 | CAGAGCAGGTACCTCCAACATCAAGGAAG |
| 6488 | CTTGCGTTTTCAGATCCAAACAGTATCCAATAGTGGTCAGTCCA |
| 6489 | TGGACTGACCACTATTGGATACTGTTTGGATCTGAAAACGCAAG |
| 6490 | GAGTAACAGTCTGAGTAGGAGAGTTGTGAGTCAAAATGTCATTTG |
| 6491 | CAAATGACATTTTGACTCACAACTCTCCTACTCAGACTGTTACTC |
| 6515 | GTTTCATGGTGGCTAGCGGATCTGACGGTTCACTAAACCAGCTCTGCTTATATAGACCT |
| 6533 | GTCAAACTTCGGACTCCATGGGTGAGGAAGATCTTCTCAGT |
| 6534 | CATTGACTCTCTCCAAGATCA |
| 6535 | ACTGAGAAGATCTTCCTCACCCATGGAGTCCGAAGTTTGAC |
| 6536 | GAGGATCTTCTTGAGCATCAACTGAGCTAGCGGATCTGACGGTTCAC |
| 6537 | GTGAACCGTCAGATCCGCTAGCTCAGTTGATGCTCAAGAAGATCCTC |
| 6538 | GTAACAGTCTGAGAAGAAGAGTTGTGAGTCAAAATGTCATTTG |
| 6539 | GACTCACAACTCTTCTTCTCAGACTGTTACTCTGGTGA |
| 6545 | TATTACTAGTTATTCCAGCTCGATCAGCAGATG |
| 6611 | GGCCTCTAGAAAGTCTCGAGGGCCGTCCGCCTTC |
| 6612 | GGTGGCTAGCCTCCCGCTCCTCCGGGTAG |
| 6613 | GGCCTCTAGAGGCCGTCGCCATATTTGGGTGTCCCAACACCTGCTGCCTG |
| 6635 | GTGAACCGTCAGATCCGCTAGCCACCATGCACAGCTGGAACTTCAAGCTGTACGTCATG |
| 6636 | GAACTTCAAGCTGTACGTCATGATGCTCAAGAAGATCCTC |
| 6637 | GAACTTCAAGCTGTACGTCATGAAATTCAAACTGAAAGAGATC |
| 6701 | CCACGAGCTAGCCACCATGCACAGCTGGAACTTCAAGCTGTACGTCATG |
| 6702 | GTTCCAGCTGTGCATGGTGGCTAGCTCGTGG |
| 6732 | GTGAACCGTCAGATCCGCTAGCTGCACACACAGATCTGGAGAGGTGAAGAATACGACCA |
| 6733 | GAGAGGTGAAGAATACGACCACCTAATATGGTGTGTAAATAAAAATAAATTACACAC |
| 6734 | GTAAATAAAAATAAATTACACACCATATTAAAACATACCAGATCTGTGTTGGTTTTTTG |
| 6735 | ACCAGATCTGTGTTGGTTTTTTGTGTGAAAGGGGGAGGGGGAGGAAAGGGGGAGGGGGA |
| 6736 | AAAGGGGGAGGGGGAGGAAAGGGGGAGGGGGAGTCGACAGCCACCATGCACAGCTGGAAC |
| 6737 | GAGAGGTGAAGAATACGACCACCTAATATAGTGTAAATAAAACACACTTACAC |
| 6738 | GTGTAAATAAAACACACTTACACTATATTAAAACATACCAGATCTGTGTTGGTTTTTTG |

**The primers are used to construct the following plasmids:**

**pZC288** (#5586, #5273, #5187, #1149), **pZC287** (#5586, #5273), **pZC289** (#3195, #5273, #3589, #3688), **pZC349** (#453, #6488, #6489, #3588), **pZC350** (#3195, #6490, #6491, #6395), **pZC379** (#6533, #4777, #6534, #6535), **pZC380** (#3195, #6536, #6537, #6538, #6539), **pZC382** (#453, #6545), **pZC400** (#3195, #6515, #6635, #6636, #6395), **pZC401** (#3195, #6515, #6635, #6637), **pZC446** (#3195, #6271, #6732, #6733, #6734, #6735, #6736, #6395), **pZC447** (#3195, #6271, #6732, #6737, #6738, #6735, #6736), **pZC414** (#6611, #6612,  #4765, #1149), **pZC415** (#6611, #6612), **pZC416** (#6613, #6702, #6701, #6395), **pZC473** ( #6701, #483).

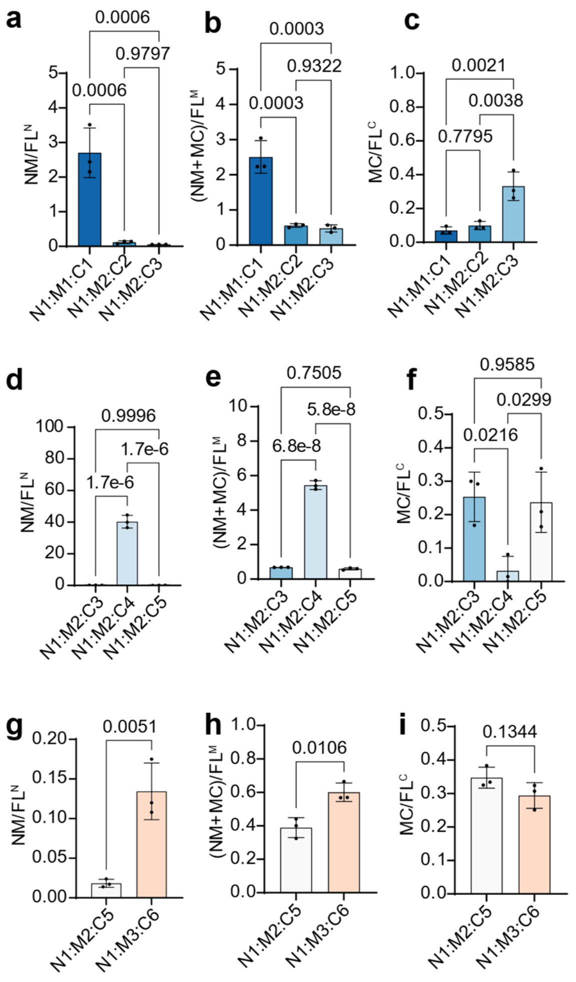


**Supplementary Fig. 1**. **Additional densitometry quantification of the Western blotting data shown in Fig. 2b, h and n**. (**a-i**) The ratio of partially assembled products versus FL dystrophin. These data were obtained from three biological repeats per condition. One-way ANOVA with Tukey's multiple comparisons test for three groups and two-tailed unpaired Student's *t* test for two groups. Source data are provided as a Source Data file.

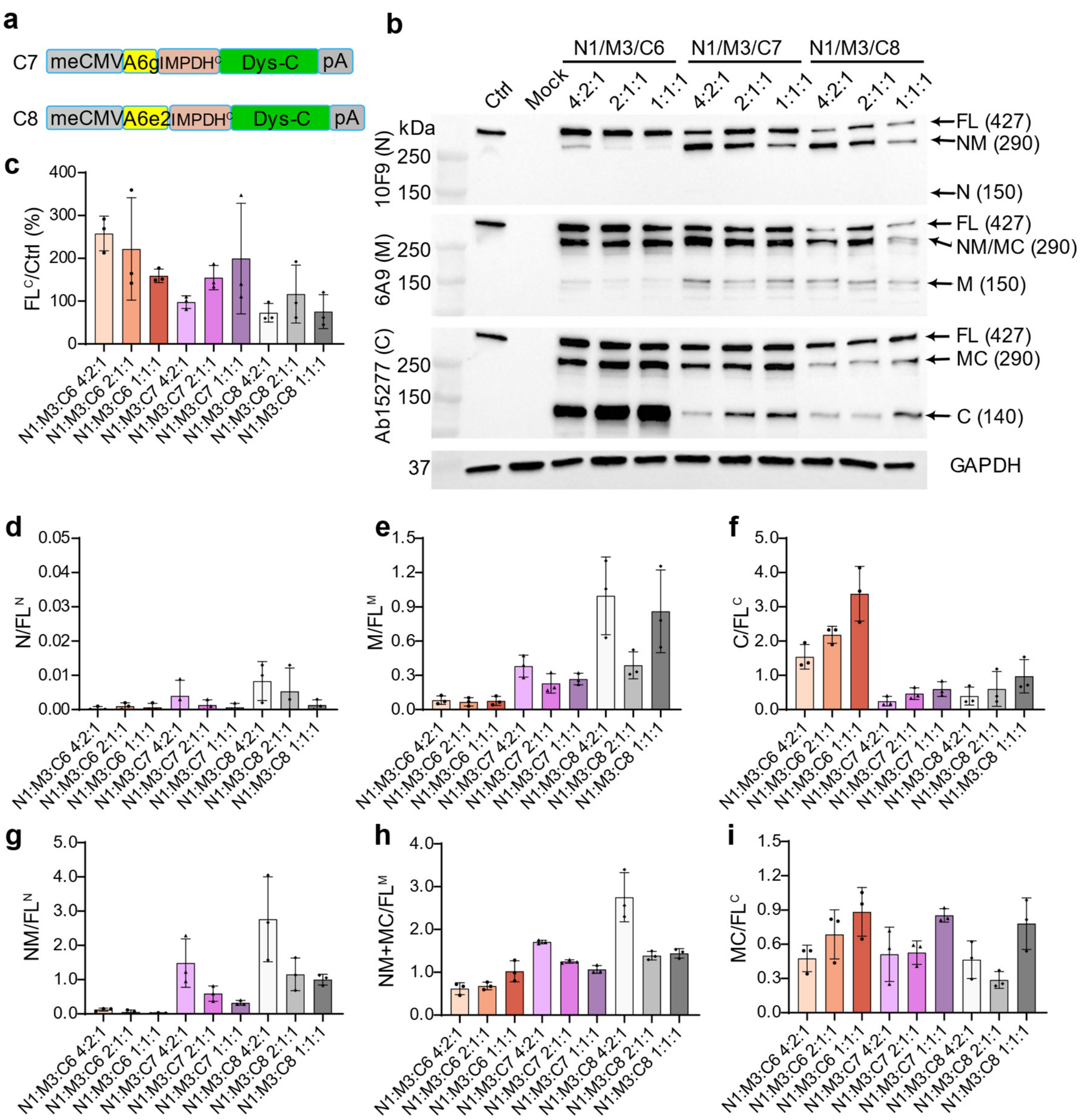

**Supplementary Fig. 2**. **Impact of poly-adenylation signals addition in the upstream of C constructs on dystrophin assembly**. (**a**) Diagram showing the C7 and C8 constructs with the position of two different poly-adenylation signals before the transgene. (**b**) Western blotting analysis of dystrophin expression in HEK293 cells transfected with or without different versions of Dys-N, M and C constructs at a molar ratio of 4:2:1. HEK293 cell lysate transfected with a FL-dystrophin construct was used as a positive control (Ctrl) and the GAPDH was used as a loading control. (**c-i**) Densitometry quantification of the FL dystrophin band intensity (**c**), the ratio of unassembled N, M or C versus FL (**d-f**), and the ratio of partially assembled products versus FL (**g-i**). These data were obtained from three biological repeats per condition. Source data are provided as a Source Data file.

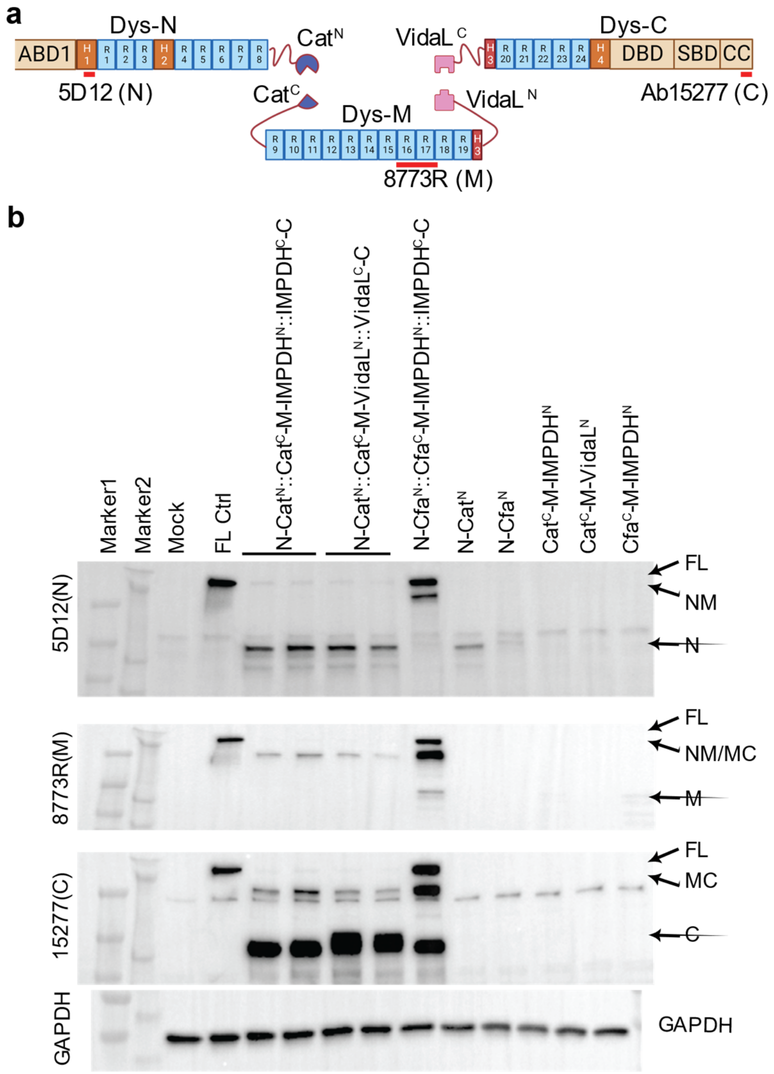


**Supplementary Fig. 3**. **Atypical split intein mediated assembly of FL-dystrophin**. (**a**) Diagram showing the domain structure of FL dystrophin, the two split sites, and the three split fragments fused with atypical intein Cat and VidaL (Dys-N, M, and C). The antigen epitopes for three different dystrophin antibodies (5D12 (N), 8773R (M) and Ab15277 (C)) were also labeled. (**b**) Western blotting analysis of dystrophin expression using the three different antibodies in HEK293 cells transfected with different dystrophin split constructs. This experiment was repeated independently for three times with similar results. Source data are provided as a Source Data file.

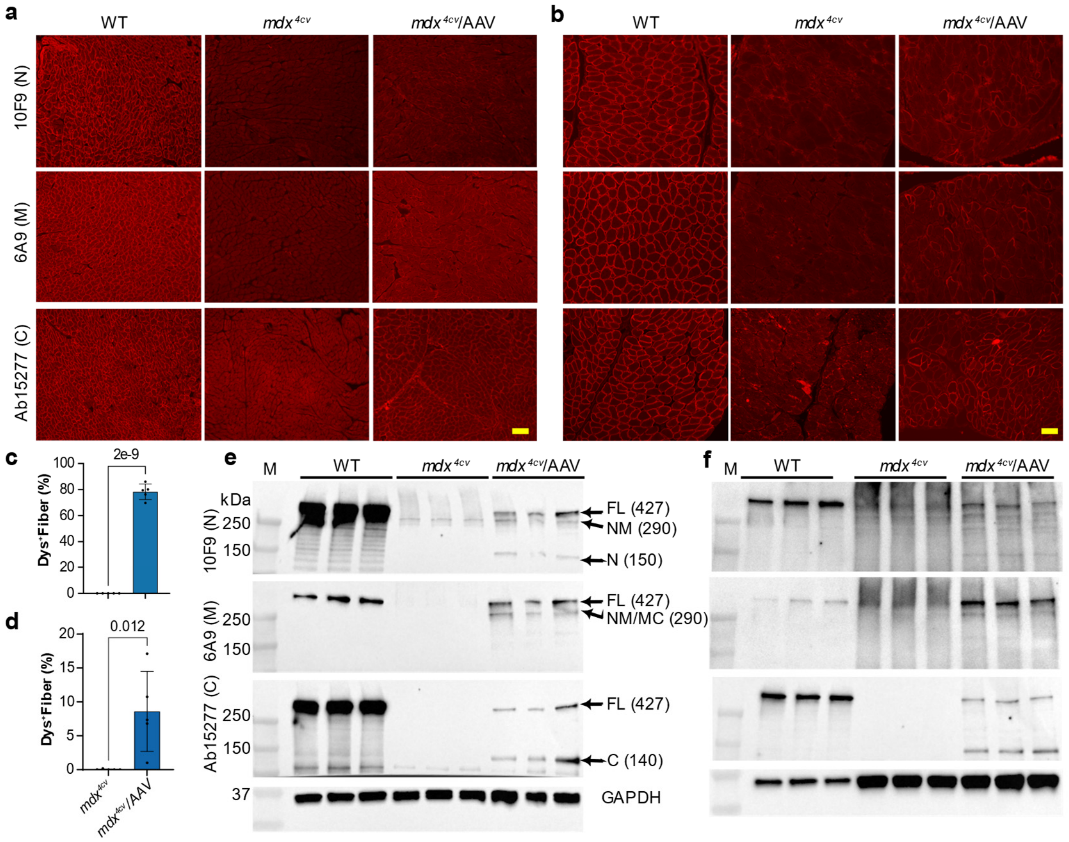


**Supplementary Fig. 4**. **Dystrophin restoration in heart and diaphragm muscle of *mdx*$^{4cv}$ following systemic MyoAAV4A-N1/M3/C6 administration**. (**a, b**) Immunofluorescence staining of heart (**a**) and diaphragm (**b**) muscle sections of WT and *mdx*$^{4cv}$ mice (n = 5 per group; 10 weeks of age) with anti-dystrophin antibodies. Scale bar: 50 µm. (**c, d**) Quantification of dystrophin-positive cardiomyocytes in heart (**c**) or muscle fibers in diaphragm muscles (**d**) of 10-week-old *mdx*$^{4cv}$ mice treated with or without AAV (n=5 per group). Two-tailed unpaired Student's *t* test. (**e, f**) Western blotting analysis of dystrophin expression in heart and diaphragm muscles from WT and *mdx*$^{4cv}$ mice treated with or without AAV (10 weeks of age, n=3 per group). Note: Six µg total proteins from WT diaphragm muscles were loaded per lane whereas thirty µg were loaded for the other lanes. Source data are provided as a Source Data file.

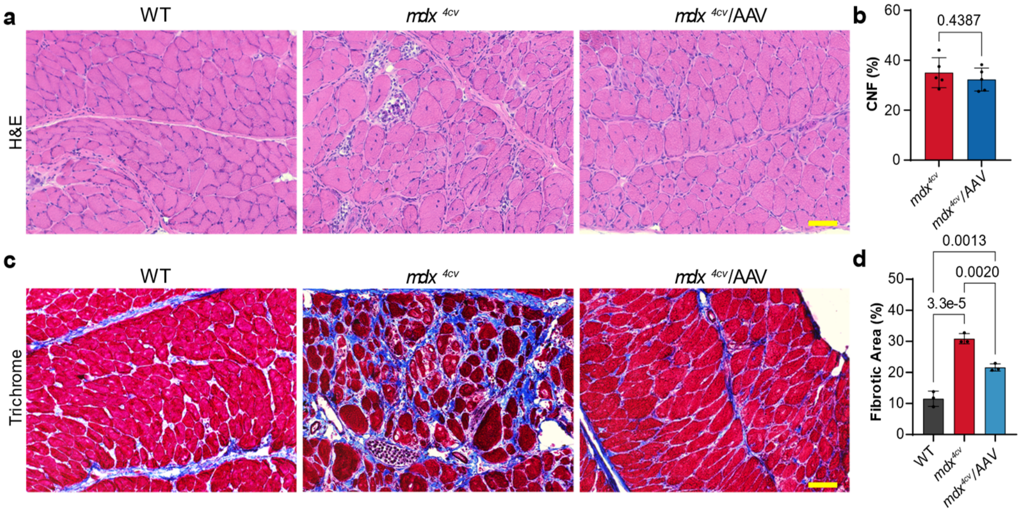


**Supplementary Fig. 5. Histopathological examination of diaphragm muscles.** (**a**) H&E staining images of diaphragm muscle sections of WT and *mdx$^{4cv}$* mice treated with or without AAV (n = 5 per group; 10 weeks of age). Scale bar: 50 µm. (**b**) Quantification of CNF in diaphragm muscles of 10-week-old *mdx$^{4cv}$* mice with or without AAV treatment (n=5 each, two-tailed unpaired *t* test). (**c**) Masson's Trichrome staining images of diaphragm muscle sections of WT and *mdx$^{4cv}$* mice treated with or without AAV (n = 3 per group; 10 weeks of age). Scale bar: 50 µm. (**d**) Quantification of percentage fibrotic area in diaphragm muscles of 10-week-old WT and *mdx$^{4cv}$* mice treated with or without AAV (n = 3 per group, one-way ANOVA with Tukey's multiple comparisons test for three groups). Source data are provided as a Source Data file.

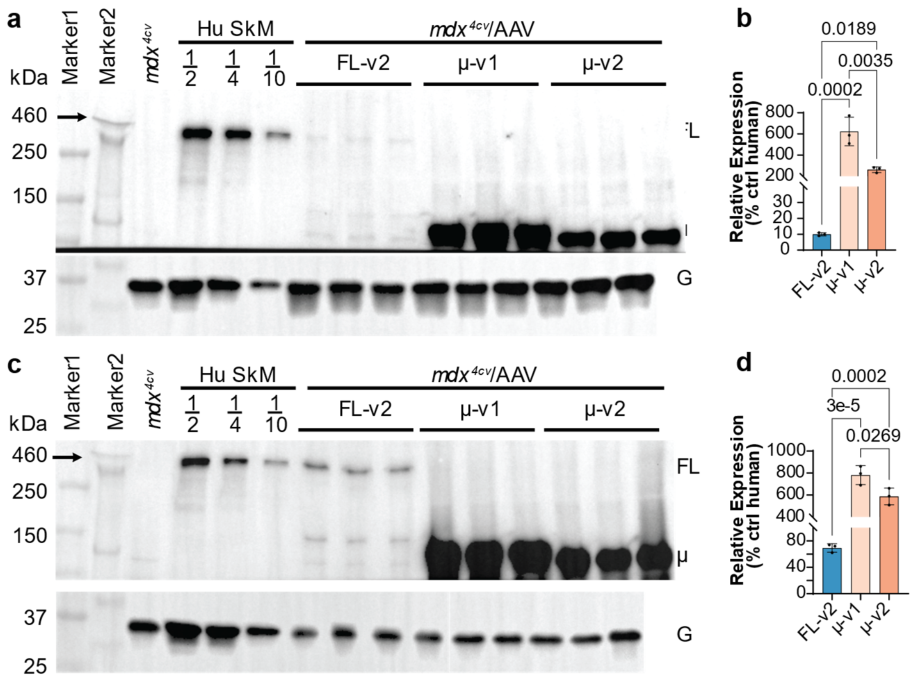


**Supplementary Fig. 6. Dystrophin restoration in diaphragm and heart muscle of *mdx*$^{4cv}$ following systemic delivery of MyoAAV4A-FL-v2, μ-v1 or μ-v2. (a-d)** Western blotting and quantification of dystrophin expression in diaphragm (**a, b**) and heart (**c, d**) muscles from WT and *mdx*$^{4cv}$ mice treated with or without AAV-FL-v2, AAV-μ-v1 or AAV-μ-v2 (10 weeks of age, n = 3 per group). Control human skeletal muscle lysate was loaded at 50% (1/2), 25% (1/4) or 10% (1/10). Source data are provided as a Source Data file.

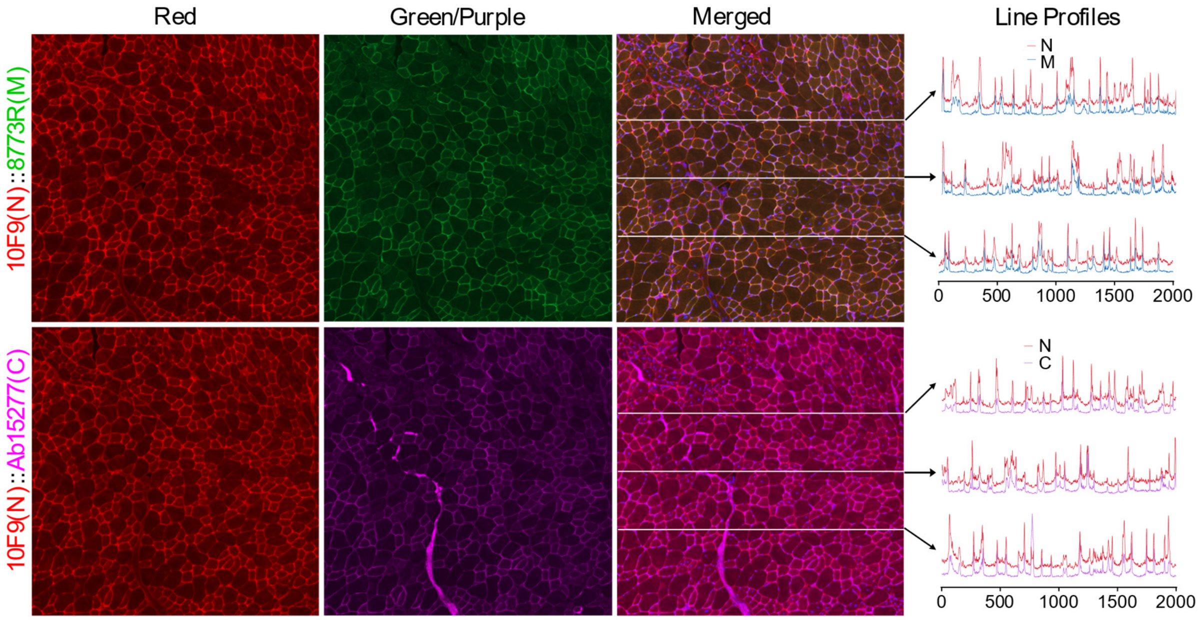


**Supplementary Fig. 7. Immunofluorescence staining analysis of dystrophin in GA muscles of *mdx$^{4cv}$* mice following systemic delivery of MyoAAV4A-FL-v2.**

Consecutive sections of GA muscles from *mdx$^{4cv}$* mice treated with MyoAAV4A-FL-v2 were co-stained with 10F9 (N, red) and 8773R (M, green) or Ab15277 (C, purple). Line profiles of fluorescence intensities were plotted for three arbitrarily selected lines. This experiment was repeated independently for three times with similar results.

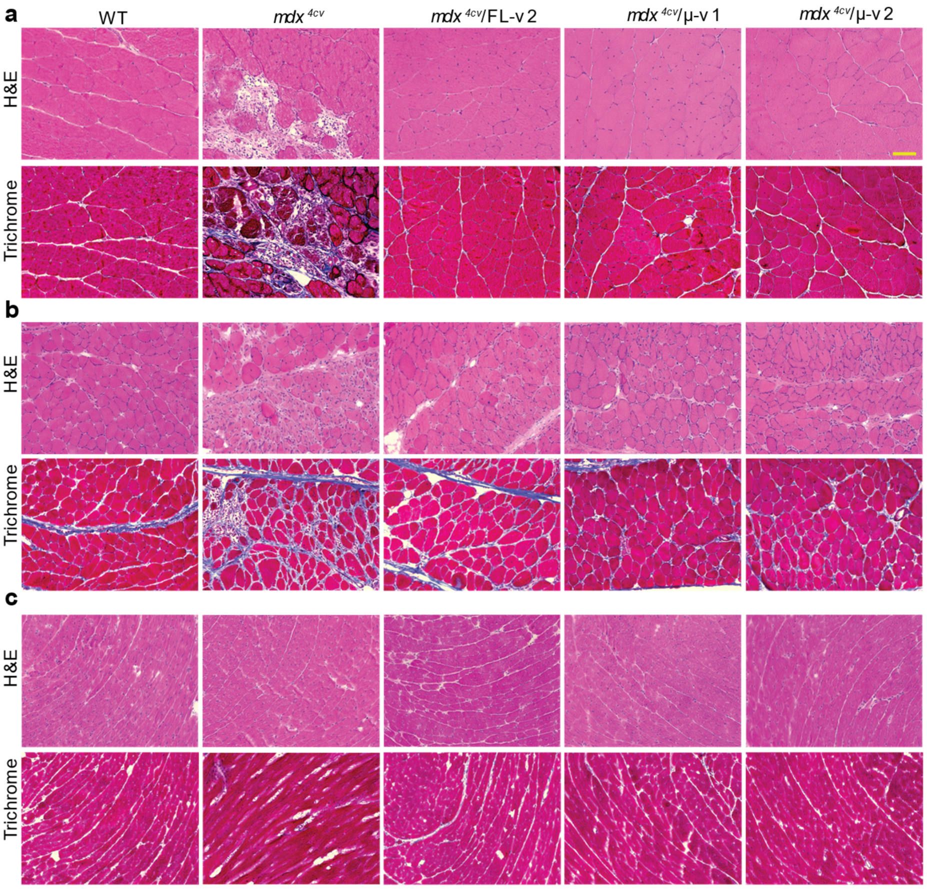


**Supplementary Fig. 8. Histological staining of GA, diaphragm and heart sections from *mdx*$^{4cv}$ mice treated with or without MyoAAV4A-FL-v2, μ-v1, or μ-v2. (a-c)**, H&E staining (upper panel) and Trichrome staining (lower panel) images of GA (a), diaphragm (b) and heart (c) sections (n = 4 per group; 10 weeks of age). Scale bar: 50 μm.
