# Supplementary material for: Systemic delivery of full-length dystrophin in Duchenne muscular dystrophy mice
Source: Nat Commun. 2024 Jul 21;15:6141. doi: 10.1038/s41467-024-50569-6 (PMC11271493; doi:10.1038/s41467-024-50569-6)
Supplement: Supplementary file 3 — Reporting Summary [file 41467_2024_50569_MOESM3_ESM.pdf]

nature portfolio

Corresponding author(s): Renzhi Han

Last updated by author(s): Jul 2, 2024

Reporting Summary

Nature Portfolio wishes to improve the reproducibility of the work that we publish. This form provides structure for consistency and transparency in reporting. For further information on Nature Portfolio policies, see our Editorial Policies and the Editorial Policy Checklist.

Statistics

For all statistical analyses, confirm that the following items are present in the figure legend, table legend, main text, or Methods section.

n/a	Confirmed	
☐	☒	The exact sample size (*n*) for each experimental group/condition, given as a discrete number and unit of measurement
☐	☒	A statement on whether measurements were taken from distinct samples or whether the same sample was measured repeatedly
☐	☒	The statistical test(s) used AND whether they are one- or two-sided<br>*Only common tests should be described solely by name; describe more complex techniques in the Methods section.*
☒	☐	A description of all covariates tested
☐	☒	A description of any assumptions or corrections, such as tests of normality and adjustment for multiple comparisons
☐	☒	A full description of the statistical parameters including central tendency (e.g. means) or other basic estimates (e.g. regression coefficient) AND variation (e.g. standard deviation) or associated estimates of uncertainty (e.g. confidence intervals)
☐	☒	For null hypothesis testing, the test statistic (e.g. *F*, *t*, *r*) with confidence intervals, effect sizes, degrees of freedom and *P* value noted<br>*Give P values as exact values whenever suitable.*
☒	☐	For Bayesian analysis, information on the choice of priors and Markov chain Monte Carlo settings
☒	☐	For hierarchical and complex designs, identification of the appropriate level for tests and full reporting of outcomes
☒	☐	Estimates of effect sizes (e.g. Cohen's *d*, Pearson's *r*), indicating how they were calculated

Our web collection on statistics for biologists contains articles on many of the points above.

Software and code

Policy information about availability of computer code

Data collection	Zeiss ZEN software v3.8 was used for epifluorescence, H&E, and Masson's trichrome image; Leica Leica SP8 Lightning confocal microscope (DMI8, Leica Microsystems, Wetzlar, Germany) equipped with Leica LAS X software (version 1.4.5.27713, Leica Microsystems, Wetzlar, Germany) was used to collect confocal fluorescence images; ChemiDoc XRS+ system was used to collect Western blot data; DMA v5.501 was used to collect muscle contractility data.
Data analysis	Graphpad prism 10.1.1 was used to analyze the data. Adobe Illustrator 28.3 was used to assemble figures. Western blots were imaged and quantified using Image Lab 6.1 software. ImageJ 1.53a was used to analysis fiber size and CNF quantification.

For manuscripts utilizing custom algorithms or software that are central to the research but not yet described in published literature, software must be made available to editors and reviewers. We strongly encourage code deposition in a community repository (e.g. GitHub). See the Nature Portfolio guidelines for submitting code & software for further information.

Data

Policy information about availability of data

All manuscripts must include a data availability statement. This statement should provide the following information, where applicable:

- Accession codes, unique identifiers, or web links for publicly available datasets
- A description of any restrictions on data availability
- For clinical datasets or third party data, please ensure that the statement adheres to our policy

All data generated or analyzed during this study are included within the article and its Supplementary Information files. Source data are provided with this paper.

Research involving human participants, their data, or biological material

Policy information about studies with human participants or human data. See also policy information about sex, gender (identity/presentation), and sexual orientation and race, ethnicity and racism.

Reporting on sex and gender	not applicable
Reporting on race, ethnicity, or other socially relevant groupings	not applicable
Population characteristics	not applicable
Recruitment	not applicable
Ethics oversight	not applicable

Note that full information on the approval of the study protocol must also be provided in the manuscript.

Field-specific reporting

Please select the one below that is the best fit for your research. If you are not sure, read the appropriate sections before making your selection.

☒ Life sciences ☐ Behavioural & social sciences ☐ Ecological, evolutionary & environmental sciences

For a reference copy of the document with all sections, see nature.com/documents/nr-reporting-summary-flat.pdf

Life sciences study design

All studies must disclose on these points even when the disclosure is negative.

Sample size	Sample size is estimated with G-power software 3.1 by the significance level (0.05), effect size (based on the data in our previous studies) and a given power (usually 0.8). We also conducted power analysis after the experiment and found enough power value (>0.8) in each statistical analysis.
Data exclusions	No data exclusion
Replication	All the experimental findings were repeated with a minimum of three independent experiments (or animals). All attempts for replication were successful.
Randomization	Mice and cells were assigned randomly into experimental groups and processed in an arbitrary order.
Blinding	All the enrolled mice or subsequent samples were labeled only with mouse ID numbers without genotype or type of treatment information. Genotype or treatment type were decoded after the data acquisition and quantification analysis were complete. The culture studies (transfection, protein isolation) were carried out identically through standard procedures that should not bias outcomes.

Reporting for specific materials, systems and methods

We require information from authors about some types of materials, experimental systems and methods used in many studies. Here, indicate whether each material, system or method listed is relevant to your study. If you are not sure if a list item applies to your research, read the appropriate section before selecting a response.

Materials & experimental systems

n/a	Involved in the study
☐	☒ Antibodies
☐	☒ Eukaryotic cell lines
☒	☐ Palaeontology and archaeology
☐	☒ Animals and other organisms
☒	☐ Clinical data
☒	☐ Dual use research of concern
☒	☐ Plants

Methods

n/a	Involved in the study
☒	☐ ChIP-seq
☒	☐ Flow cytometry
☒	☐ MRI-based neuroimaging

Antibodies

Antibodies used

Mouse anti-MANHINGE1B (10F9) Developmental Studies Hybridoma Bank WB (1:100);
Mouse anti-MANHINGE1C (5D12) Developmental Studies Hybridoma Bank WB (1:100);
Mouse anti-MANEX50 (6A9) Developmental Studies Hybridoma Bank WB (1:100);
Mouse anti-α-dystroglycan (IIH6 C4) Developmental Studies Hybridoma Bank IF (1:10);
Mouse anti-β-dystroglycan Santa Cruz Biotechnology #sc-33702 IF (1:50);
Mouse anti-α-sarcoglycan Abcam #ab234589 IF (1:100);
Mouse anti-β-sarcoglycan Santa Cruz Biotechnology #sc-14176 IF (1:50);
Mouse anti-nNOS Santa Cruz Biotechnology #sc-5302 IF (1:50);
Mouse anti-α-dystrobrevin Becton Dickinson and Company #610766 IF (1:100)
Mouse anti-p44/42 MAPK (Erk1/2) Cell Signaling Technology #4696 WB (1:1000)
Rabbit anti-Phospho-p44/42 MAPK (Erk1/2) Cell Signaling Technology #9101 WB (1:500)
Rabbit anti-MURC Abcam #ab121647 IF (1:100)
Rabbit anti-DMD/8773R NeoBiotechnologies WB (1:200);
Rabbit anti-Dystrophin Abcam #ab15277 WB (1:1000);
Rabbit anti-GAPDH Cell Signaling Technology #2118S WB (1:2000);
Rat anti-laminin-α2 Enzo Life Sciences #ALX-804-190-C100 IF (1:100);
Goat anti-Mouse lgG (H+L) Alexa Fluor'M 594 lnvitrogen #A-11032 IF(1:400);
Goat anti-Rat lgG (H+L) Alexa Fluor'M 488 lnvitrogen #A-11006 IF(1:400);
Goat anti-Rabbit lgG (H+L) Alexa Fluor Texas Red lnvitrogen #T2767 IF(1:400);
Goat anti-Mouse lgM (H+L) Alexa Fluor'M 568 lnvitrogen #A21043 IF(1:400);
Secondary HRP-conjugated goat anti-mouse Cell Signaling Technology#7076 WB (1:4000);
Secondary HRP-conjugated goat anti-rabbit Cell Signaling Technology# 7074 WB (1:4000).

Validation

All antibodies are available on the manufacturer's websites as listed below. The antibodies have been validated by the manufacturers. No additional validation was carried out.

1. Mouse anti-MANHINGE1B (10F9) Developmental Studies Hybridoma Bank
The species: Canine, Human
Application: WB
Website: https://dshb.biology.uiowa.edu/MANHINGE1B-clone-10F9

2. Mouse anti-MANHINGE1C (5D12) Developmental Studies Hybridoma Bank
The species: Canine, Human
Application: WB
Website: https://dshb.biology.uiowa.edu/MANHINGE1C-5D12

3. Mouse anti-MANEX50 (6A9) Developmental Studies Hybridoma Bank
The species: Human
Application: WB
Website: https://dshb.biology.uiowa.edu/MANEX50-6A9

4. Mouse anti-α-dystroglycan (IIH6 C4) Developmental Studies Hybridoma Bank
The species: Mus musculus (House mouse), Homo sapiens (Human)
Application: WB, IHC, IF, IHC-IF, FC/FACS, IHC-Frozen
Website: https://dshb.biology.uiowa.edu/IIH6-C4

5. Mouse anti-β-dystroglycan Santa Cruz Biotechnology #sc-33702
The species: mouse, rat, human
Application: WB, IP, IF and IHC(P)
Website: https://www.scbt.com/p/beta-dystroglycan-antibody-4f7?requestFrom=search

6. Mouse anti-α-sarcoglycan Abcam #ab234589
The species: Mouse, Rat, Human
Application: IHC-Fr
Website: https://www.abcam.com/products/primary-antibodies/alpha-sarcoglycan-antibody-ivd3-a9-ab234589.html

7. Mouse anti-β-sarcoglycan Santa Cruz Biotechnology #sc-14176
The species: mouse, rat and human

Application: WB, IF, ELISA
Website: extension://bfdogplmndidlpjfhoijckpakkdjkkil/pdf/viewer.html?file=https%3A%2F%2Fdatasheets.scbt.com%2Fsc-14176.pdf

8. Mouse anti-nNOS Santa Cruz Biotechnology #sc-5302
The species: mouse, rat and human
Application: WB, IP, IF and ELISA
Website: https://www.scbt.com/p/nos1-antibody-a-11?requestFrom=search

9. Mouse anti-α-dystrobrevin Becton Dickinson and Company #610766
The species: Rat, Mouse, Rabbit
Application: WB, IHC, IF
Website: https://www.bdbiosciences.com/en-us/products/reagents/microscopy-imaging-reagents/immunohistochemistry-reagents/purified-mouse-anti-dystrobrevin.610766

10. Mouse anti-p44/42 MAPK (Erk1/2) Cell Signaling Technology #4696
The species: H M R Mk Mi Z B Pg
Application: WB, IF, IHC
Website:https://www.cellsignal.com/products/primary-antibodies/p44-42-mapk-erk1-2-l34f12-mouse-mab/4696

11. Rabbit anti-Phospho-p44/42 MAPK (Erk1/2) Cell Signaling Technology #9101
The species: H M R Hm Mk Mi Dm Z B Pg Ce
Application: WB, IF, IHC, F
Website: https://www.cellsignal.com/products/primary-antibodies/phospho-p44-42-mapk-erk1-2-thr202-tyr204-antibody/91011

12.Rabbit anti-MURC Abcam #ab121647 IF (1:100)
The species: Human
Application: ICC/IF, IHC-P
Website:https://www.abcam.com/products/primary-antibodies/murc-antibody-ab121647.html

13. Rabbit anti-DMD/8773R NeoBiotechnologies
The species: Human
Application: IF, IHC, WB
Website: https://www.neobiotechnologies.com/product/dystrophin-dmd-marker-of-duchenne-and-becker-muscular-dystrophy-9/

14. Rabbit anti-Dystrophin Abcam #ab15277
The species: Mouse, Human
Application: IHC-Fr, IHC-P
Website: https://www.abcam.com/products/primary-antibodies/dystrophin-antibody-ab15277.html

15. Rabbit anti-GAPDH Cell Signaling Technology #2118S
The species: H M R Mk B Pg
Application: WB, IF, IHC
Website: https://www.cellsignal.com/products/primary-antibodies/gapdh-14c10-rabbit-mab/2118

16. Rat anti-laminin-α2 Enzo Life Sciences #ALX-804-190-C100
The species: Human, Mouse
Application: ELISA, IHC, IP
Website: https://www.fishersci.com/shop/products/laminin-2-a-2-chain-4h8-2-100/NC1425153

Eukaryotic cell lines

Policy information about cell lines and Sex and Gender in Research

Cell line source(s)	HEK293 cell line was obtained from the American Type Culture Collection (ATCC).
Authentication	HEK293 cell line was not authenticated.
Mycoplasma contamination	HEK293 cells were tested for mycoplasma contamination by PCR.
Commonly misidentified lines (See ICLAC register)	To the best of our knowledge, no misidentified cell lines have been used in this study.

Animals and other research organisms

Policy information about studies involving animals; ARRIVE guidelines recommended for reporting animal research, and Sex and Gender in Research

Laboratory animals	The C57BL/6J and mdx4cv (B6Ros.Cg-Dmd mdx4cv/J) mice purchased from the Jackson Laboratory and housed at Indiana University Laboratory Animal Resource Center following animal use guidelines. The mice from 3-12 weeks of age were used for experiments. All mice were maintained under standard conditions of constant temperature (72±4°F), humidity (relative, 30–70%), in a specific

pathogen-free facility and exposed to a 12-h light/dark cycle.

Wild animals	This study did not involve wild animals.
Reporting on sex	This study used male mice.
Field-collected samples	This study did not involve samples collected from the field.
Ethics oversight	The animal experiments were ethically reviewed by the Animal Care, Use and Review Committee of Indiana University and carried out in accordance with animal use guidelines.

Note that full information on the approval of the study protocol must also be provided in the manuscript.

Plants

Seed stocks	N/A
Novel plant genotypes	N/A
Authentication	N/A
